# EANM procedure guideline for the treatment of liver cancer and liver metastases with intra-arterial radioactive compounds

**DOI:** 10.1007/s00259-021-05600-z

**Published:** 2022-02-11

**Authors:** M. Weber, M. Lam, C. Chiesa, M. Konijnenberg, M. Cremonesi, P. Flamen, S. Gnesin, L. Bodei, T. Kracmerova, M. Luster, E. Garin, K. Herrmann

**Affiliations:** 1 Department of Nuclear medicine, University clinic Essen, Essen, Germany; 2 Department of Radiology and Nuclear Medicine, University Medical Center Utrecht, Heidelberglaan 100, 3584 CX Utrecht, The Netherlands; 3 Nuclear Medicine, Foundation IRCCS National Tumour Institute, Milan, Italy; 4 Nuclear Medicine Department, Erasmus MC, Rotterdam, The Netherlands; 5 Radiation Research Unit, IEO European Institute of Oncology IRCCS, Via Giuseppe Ripamonti, 435, 20141 Milan, MI Italy; 6 Department of Nuclear Medicine, Institut Jules Bordet-Université Libre de Bruxelles (ULB), 1000 Brussels, Belgium; 7 Institute of Radiation physics, Lausanne University Hospital, University of Lausanne, Lausanne, Switzerland; 8 Molecular Imaging and Therapy Service, Department of Radiology, Memorial Sloan Kettering Cancer Center, New York, USA; 9 Department of Medical Physics, Motol University Hospital, Prague, Czech Republic; 10 Department of Nuclear medicine, University hospital Marburg, Marburg, Germany; 11 Department of Nuclear Medicine, Cancer, Institute Eugène Marquis, Rennes, France

**Keywords:** Guidelines, Nuclear medicine, Liver cancer, ^90^Y-microspheres, SIR-spheres®, TheraSphere®, Resin microspheres, Glass microspheres, ^166^Ho-microspheres, QuiremSpheres®, Dosimetry

## Abstract

**Abstract:**

Primary liver tumours (i.e. hepatocellular carcinoma (HCC) or intrahepatic cholangiocarcinoma (ICC)) are among the most frequent cancers worldwide. However, only 10–20% of patients are amenable to curative treatment, such as resection or transplant. Liver metastases are most frequently caused by colorectal cancer, which accounts for the second most cancer-related deaths in Europe. In both primary and secondary tumours, radioembolization has been shown to be a safe and effective treatment option. The vast potential of personalized dosimetry has also been shown, resulting in markedly increased response rates and overall survival. In a rapidly evolving therapeutic landscape, the role of radioembolization will be subject to changes. Therefore, the decision for radioembolization should be taken by a multidisciplinary tumour board in accordance with the current clinical guidelines. The purpose of this procedure guideline is to assist the nuclear medicine physician in treating and managing patients undergoing radioembolization treatment.

**Preamble:**

The European Association of Nuclear Medicine (EANM) is a professional non-profit medical association that facilitates communication worldwide among individuals pursuing clinical and research excellence in nuclear medicine. The EANM was founded in 1985. These guidelines are intended to assist practitioners in providing appropriate nuclear medicine care for patients. They are not inflexible rules or requirements of practice and are not intended, nor should they be used, to establish a legal standard of care. The ultimate judgment regarding the propriety of any specific procedure or course of action must be made by medical professionals taking into account the unique circumstances of each case. Thus, there is no implication that an approach differing from the guidelines, standing alone, is below the standard of care. To the contrary, a conscientious practitioner may responsibly adopt a course of action different from that set out in the guidelines when, in the reasonable judgment of the practitioner, such course of action is indicated by the condition of the patient, limitations of available resources or advances in knowledge or technology subsequent to publication of the guidelines. The practice of medicine involves not only the science but also the art of dealing with the prevention, diagnosis, alleviation and treatment of disease. The variety and complexity of human conditions make it impossible to always reach the most appropriate diagnosis or to predict with certainty a particular response to treatment. Therefore, it should be recognised that adherence to these guidelines will not ensure an accurate diagnosis or a successful outcome. All that should be expected is that the practitioner will follow a reasonable course of action based on current knowledge, available resources and the needs of the patient to deliver effective and safe medical care. The sole purpose of these guidelines is to assist practitioners in achieving this objective.

## Purpose

The purpose of this guideline is to assist nuclear medicine physicians in:

1. Patient selection: evaluating patients who might be candidates for treatment using intra-arterial radioactive microspheres for primary or secondary liver cancer.
2. Treatment procedures: providing information on treatment methods.
3. Clinical follow-up: understanding and evaluating efficacy and toxicity.

These guidelines represent an update of the 2011 EANM guidelines on the treatment of liver cancer and liver metastases with intra-arterial radioactive compounds. Information still considered to be up to date was retained. Due to the limited use of ^131^I-lipiodol in Europe, no further update will be provided on this specific treatment modality. In the light of recent trials, the main focus of this update will instead be placed on dosimetric concepts in different treatment scenarios and the use of the newly introduced ^166^Ho-microspheres.

## Background information and definitions

### Definitions

#### Radionuclides

^90^Y is a beta-emitting radionuclide with a physical half-life of 2.67 days (64.2 h), without emission of gamma photons but with the emission of secondary “bremsstrahlung” photons. ^90^Y also emits positrons in 32 decays over a million. Despite this scarcity, ^90^Y PET imaging is routinely performed post-treatment. The maximum and mean beta particle energies are 2.28 and 0.94 MeV, respectively. The maximum and mean ranges in soft tissue are 11 and 4 mm, respectively [1].

^166^Ho is a beta-emitting radionuclide with a physical half-life of 1.1 days (26.8 h). The beta particle energies include 1.85 MeV (50.0%) and 1.77 MeV (48.7%). The maximum and mean ranges in soft tissues are 8.7 and 2.2 mm, respectively. ^166^Ho emits gamma photons at 80 keV (yield 6.7%) and 1.4 MeV (yield 0.9%).

^99m^Tc is a metastable nuclear isomer of ^99^Tc with a half-life of 6.0 h. It emits gamma photons with a photon energy of 140 keV.

#### Radioactive microspheres (Table 1)

Resin microspheres are made of an acrylic polymer with a median size of 30 μm in diameter, in which ^90^Y is bound to the carboxylic group on the surface of the polymer after production of microspheres.

**Table 1:** Radioembolization microspheres characteristics

Characteristics	SIR-Spheres®	TheraSphere®	QuiremSpheres®
Material	Resin	Glass	Poly-L-lactic acid
Particle size and range (μm)	30 (20–60)	25 (20–30)	30 (15–60)
Embolic effect	Moderate	Mild	Moderate
Activity per sphere (Bq)	40–70	4534 *	200–400
Specific gravity (g/dL)	1.6	3.7	1.4
Activity available (GBq)	3^#^	3–20^ “	“
Handling for dispensing	Required	Not required	Not required
Multiple dosing from one vial	Possible	Not possible	Not possible

Modified from Salem and Thurston [2], Smits [3] and Westcott [4]

*Direct measure by Pasciak et al. [5] at calibration, the IFU provide a value of 2500 Bq. The value is variable according to physical decay depending on the day and time of treatment

^#^Prescribed activity should be withdrawn on site. The FLEXdose option allows injection 3 days before calibration, when the vial activity is 10 GBq

^Vials of 3–20 GBq in steps of 0.5 GBq, calibrated at noon on the Sunday before treatment with a shelf-life of 12 days

“Patient-specific activity is calibrated at the day and time of treatment

Glass microspheres are made of glass with a median size of 25 μm, in which ^89^Y, embedded in the glass matrix, is activated to ^90^Y in a nuclear reactor.

^166^Ho-microspheres are made of poly-L-lactic acid with a median size of 30 μm, in which ^165^Ho, embedded in the matrix, is activated to ^166^Ho by neutron activation in a nuclear reactor.

^99m^Tc-macroaggregated albumin (MAA) particles mostly have a size between 10 and 100 μm; ^99m^Tc-human serum albumin (HSA) particles mostly have a size between 15 and 50 μm. Both are synthesized by labeling of MAA and HSA-kits with ^99m^Tc, respectively.

## Background

The treatment of hepatic malignancies via the hepatic arterial route is based on the existence of arterial tumoural hypervascularization. Tumou﻿rs bigger than 2 cm in diameter draw more than 80% of their blood supply from the hepatic artery. Normal liver parenchyma draws more than 80% of blood from the portal vein. Highly selective tumour targeting can thus be achieved by delivery of radioactive microspheres into the hepatic artery [6–8].

Unlike hepatocellular carcinoma (HCC), liver metastases may have variable vascularity, from avascular hepatic cysts to normal hepatic parenchyma, less vascularised metastatic lesions (e.g. colon, pancreas, breast) and finally hypervascular metastases (e.g. renal, neuroendocrine, thyroid).

In ^90^Y-microspheres radioembolization, pre-treatment intra-arterial ^99m^Tc-labelled albumin macroaggregated albumin^1^ (^99m^Tc-MAA) scintigraphy^2^ (see below) is mandatory to quantify potential liver-lung shunting and to exclude reflux to bowel, stomach or pancreas [9, 10]. Note that the use of ^99m^Tc-MAA (although accepted worldwide) is in principle off-label, since its use is indicated only for lung perfusion scintigraphy. In ^166^Ho-microspheres radioembolization, the administration of a scout dose of ^166^Ho-microspheres (i.e. 250 MBq) has been shown to be safe and more accurate for the calculation of the lung shunt fraction when compared to ^99m^Tc-MAA [11, 12]. The therapeutic effect of radioembolization is essentially driven by a radiation effect, as opposed to the ischaemia associated with chemoembolization or bland embolization. The radiobiological effect results from beta particle irradiation, which causes delayed death of tumour cells surrounding microvessels containing a high radioactive microspheres concentration [13–19].

One of the main differences between the different microspheres is the specific activity of each microsphere at the calibration time, being much higher for glass microspheres (i.e. approximately 4500 Bq/microsphere at calibration), in contrast to approximately 340 Bq/microsphere for ^166^Ho-microspheres and 50 Bq/microsphere for resin microspheres.

Commercially available vials of glass microspheres contain up to 20 GBq, ^166^Ho-microspheres up to approximately 15 GBq and resin microspheres 10 GBq fixed. Consequently, at the same prescribed activity, glass microspheres have the least embolic effect, being injected in much lower numbers. Potentially, at the same prescribed activity, the higher number of resin microspheres may provide a more uniform dose distribution, with a higher biological effect (i.e. toxicity and efficacy), with ^166^Ho-microspheres falling in between. This argument holds if the clustering effect is neglected. Being present in both kinds of ^90^Y-microspheres [16], the optimal number of microspheres to obtain uniform coverage is still unclear [20]. A theoretical disadvantage of glass microspheres is the often quoted, but never demonstrated influence of gravity on their biodistribution [21–24]. Resin microspheres are also available at higher specific activity, the so-called 1-, 2- or 3-day calibration, with 3 GBq activity calibrated at day 0, but supplied days before to increase specific activity.

### Indication

Primary or secondary liver tumours. With mounting evidence, the indication with regard to tumour type and specific clinical settings will rapidly change over the years. This falls outside the scope of this procedural guideline.

### Contraindications

*Absolute*

- Pregnancy, breastfeeding
- Life expectancy of less than 3 months
- Clinical liver failure (i.e. ascites, icterus, encephalopathy)
- Disseminated extrahepatic malignant disease (see section Diagnostic work-up for reference)
- In case the pre-treatment intra-arterial scout dose scintigraphy (or peri-procedural C-arm CT) shows any extrahepatic activity (or contrast enhancement) in the gastrointestinal tract that cannot be corrected by angiographic techniques (exceptions include the gallbladder, lymph nodes, falciform ligament)

*Relative*

- Child-Pugh score higher than B7. A liver decompensation rate as high as 89% after glass administration with standard dosage (i.e. single compartment modelling) has been reported in patients with B7 liver cirrhosis [68]. Caution is warranted in any treatment that is not (bi-)segmental.
- High intrahepatic tumour burden. Depending on tumour type, more (e.g. neuroendocrine, more indolent, hypervascular, symptomatic disease in need of palliation) or less (hepatocellular carcinoma, underlying liver disease, more aggressive) tumour burden is acceptable. A cut-off of 50–70% is often reported.
- High extrahepatic tumour burden. Depending on tumour type, more (e.g. neuroendocrine, prognosis depends on liver disease, more indolent) or less (e.g. intrahepatic cholangiocarcinoma (ICC), more aggressive) extrahepatic disease is acceptable. Hilar lymph nodes (up to 2 cm short axis) and lung nodules (up to 1 cm; up to 5) are often accepted.
- Main portal vein thrombosis (PVT) with poor targeting evidenced by scintigraphy. These patients will have a very poor outcome with little benefit from treatment.
- Poor targeting of portal vein thrombosis in the main trunk.
- Acute or severe chronic renal failure (i.e. creatinine clearance <30 ml/min).
- Contraindications to hepatic artery catheterization (e.g. unmanageable coagulation disorder, renal failure, severe allergy to contrast media, vascular abnormalities).
- Lung shunting that would lead to a lung dose >30 Gy per session or > 50 Gy cumulatively (determined by pre-treatment planar ^99m^Tc-MAA scintigraphy). Not an absolute contraindication, because lung shunting by planar ^99m^Tc-MAA is overestimated and a reliable safety limit has not been established yet.

*Special warnings*

- Inadvertent delivery of microspheres to the gastrointestinal tract or pancreas may cause acute abdominal pain, acute pancreatitis or peptic ulceration.
- Prior external beam radiation therapy (EBRT) of the liver. Radioembolization appears to be safe for the treatment of hepatic malignancies only in patients who have had limited hepatic exposure to prior EBRT, with the strongest predictor for hepatotoxicity being the liver fraction exposed to ≥30 Gy [25]. While liver regeneration might allow combined treatments, little is known about the cumulative thresholds. Absorbed dose values cannot be simply summed, since a reliable method to combine such values is still lacking.
- Repeated radioembolization treatments seem less critical than radioembolization after EBRT. Young et al. [26] repeated lobar radioembolization an average of 2.6 times in HCC patients with 17% toxicity incidence. No data is available to strictly demonstrate whether the dose tolerance in a second treatment can be considered the same as for the first treatment. However, liver regeneration might allow this strategy, provided that a sufficient interval between treatments is kept (3 months or longer).
  Radioembolization after peptide radioreceptor therapy (PRRT) of neuroendocrine tumours was reported as safe and effective, with a low incidence (5%) of REILD [27, 28].
- Markedly abnormal liver function tests (e.g. aspartate aminotransferase or alanine aminotransferase >5 times upper limit of normal or elevated bilirubin). Caution is advised when bilirubin exceeds normal levels, especially in bilobar metastatic disease, in case whole liver treatment is warranted.
- Excessive radiation to the normal liver parenchyma may result in radiation hepatitis or radioembolization-induced liver disease [29], characterized by the combination of increased bilirubin, low albumin and ascites. The usual onset is 2–6 months after radioembolization [79]. It may co-exist with progressive disease (complicating the clinical presentation), but the diagnosis is definite in the absence of progressive disease.
- The risk of cholangitis or abscess may be elevated in patients with a history of biliary intervention. Although a direct relation has not been established (in contrast to TACE), caution is advised, and prophylactic antibiotics may be considered.
- Excessive hepatotoxic systemic therapy prior to radioembolization may increase the risk of post-treatment liver failure. However, in mCRC, it was evidenced in randomized controlled trials that the combination of first- and second-line systemic therapy with radioembolization is safe [30–32]. In HCC, the combination of first-line sorafenib and radioembolization proved to be safe [33], but an unacceptable risk of liver failure was identified for ICC patients with underlying cirrhosis treated as first-line with concomitant radioembolization and standard chemotherapy in a multicentre phase II study [34].
- Non-dosimetric activity calculation methods (single compartment modelling; BSA-method) may lead to under- or overtreatment. Please note that under- and overtreatment may be equally harmful to the patient.

## Diagnostic work-up

### Clinical and laboratory evaluation

Patients should be accurately staged according to international standards. Clinical history, physical examination, laboratory values and performance status are evaluated. Parameters to assess the indication for radioembolization include determination of tumour load, volume and serum tumour markers (e.g. alpha-fetoprotein (AFP), carcinoembryonic antigen (CEA)). Serum liver enzymes, bilirubin, albumin, cholinesterase, blood cell count, coagulation and creatinine should be monitored and known before the procedure. Selection of patients with adequate hepatic reserve and good functional status will maximize the beneficial therapeutic effect with minimal risk to normal liver parenchyma.

### Pre-treatment imaging

Pre-treatment imaging is important to establish feasibility and objectives of treatment, which can extend from palliative treatments to radiation segmentectomy for very limited disease. Imaging techniques include contrast-enhanced CT or MRI performed within 30 days of the procedure for the calculation of the tumour volume and for staging purposes. In addition, (early) arterial phase CT is done to identify hepatic arterial anatomy (e.g. origin of right gastric artery, origin of segment 4 artery). Also, for FDG-avid hepatic malignancies, ^18^F-fluorodeoxyglucose (FDG) PET is performed for staging purposes, to identify metabolically active liver disease and to rule out prognostically relevant extrahepatic disease. Moreover, metabolic response can be seen 4–6 weeks after radioembolization, whereas response at cross-sectional anatomic imaging may take longer (e.g. 2–3 months) [35, 36]. Both pre- and early post-therapeutic FDG-PET have shown potential to stratify patients with regard to OS and PFS [37–40]. Pre-therapeutic hepatobiliary scintigraphy (e.g. using ^99m^Tc-mebrofenin) may be considered, particularly in patients scheduled for partial liver treatment, to measure segmental distribution of liver function and quantify functional liver remnants [41].

There is consensus that radioembolization is generally only indicated in scenarios with no or very limited extrahepatic spread; however, different definitions of limited extrahepatic spread have been employed [30, 42–46]. In an analysis of over 1000 patients with primary and metastatic liver neoplasms prospectively included in the observational study ‘CIRSE Registry for SIR-Spheres® Therapy (CIRT)’, low overall survival (OS) was mainly associated with extrahepatic disease extent [46]. In HCC, a sub-analysis of the SORAMIC trial study cohort did not show a negative impact of limited extrahepatic spread, which was defined as involvement of lymph nodes, bones and/or adrenal glands [47]. In mCRC, up to five pulmonary nodules *and* either lymph nodes belonging to one region [30] or one additional metastatic site amenable to future definitive treatment [43] have been employed as thresholds for limited extrahepatic spread and may be used for reference.

In patients with ICC, lymph node metastases have not shown any negative impact on OS and may therefore not be viewed as exclusion criteria [48]. In the presence of solid organ metastases, caution is warranted, and treatment should be decided on a case-by-case basis depending on whether the intra- or extrahepatic tumour spread is considered life-limiting.

### Peri-procedural imaging

Peri-procedural angiography, performed by high-speed multislice CT (angio-CT), cone-beam CT, or digital subtraction angiography, is valuable for procedure planning, in order (1) to assess the hepatic vascular anatomy, (2) to verify the presence or absence of portal venous thrombosis in the portal venous phase, and the possible presence of major arteriovenous malformations or aberrant vasculature, (3) to assess perfused volumes and tumour targeting, (4) to assess intrahepatic interval progression and potential new lesions, and (5) to assess extrahepatic contrast enhancement, precluding safe administration of radioactive microspheres.

99mTc-MAA scintigraphy / 166Ho-microspheres scout dose9

9mTc-MAA is a surrogate for the estimated activity distribution of 90Y- and 166Ho-microspheres. Intrinsic differences in size and rheological properties between MAA [49] and microspheres, together with the operator dependent positioning of the catheter tip in the therapeutic session, are responsible for documented major variations in a minority of patients between MAA predicted and actual therapeutic distribution [50]. Scintigraphy should be performed as soon as possible (preferably within one hour) after administration because of the degradation of 99mTc-MAA (timing of preparation is of less importance); 99mTc-human serum albumin is more resistant to degradation, but also less available [51]. A total of approximately 150 MBq of 99mTc-MAA is administered into the respective branch (or branches) of the hepatic artery.

Planar imaging is used for the first liver-lung shunt calculation. The estimate without attenuation correction gives a large overestimation in comparison with attenuation-corrected evaluations [12, 52]. Scatter correction could also be important, especially for the right lung, strongly influenced by liver photon emission. However, since tolerance doses for the lungs were empirically established using planar imaging, lung doses must be primarily calculated with this approach [53–55]. For more accurate calculation, an attenuation and scatter corrected tomographic SPECT/CT covering lung is suggested in cases of substantial lung shunt fraction (> 10%), both pre- and post-treatment.

In the case of multifocal HCC, liver-lung shunting may be assessed before each treatment at the lobar level, because tumours located in different lobes may shunt to varying degrees. The lung shunt fraction is determined by the following equation:

$$\mathrm{Lung}\ \mathrm{shunt}\ \mathrm{fraction}=\frac{ lung\ counts}{lung+ liver\ counts}$$

SPECT (or SPECT/CT) centred on the upper abdomen is advised for the assessment of gastrointestinal shunting,^3^ appreciation of tumour targeting and better visualization. Moreover, obtained images allow for dosimetric evaluation.

Pre-treatment ^99m^Tc-MAA targeting predicts the ^90^Y absorbed dose to the non-tumour and (less accurately) to the tumour tissue [56, 57]. It can be used for personalized dosimetry and treatment planning, even if some reports have shown poor prediction of post-treatment ^90^Y-microsphere distribution by ^99m^Tc-MAA [58–60].

Indeed, many studies have demonstrated a clear dose–response relationship based on ^99m^Tc-MAA dosimetry a least for HCC using both glass [61–64] or resin microspheres [65, 66].

Most importantly, level 1 evidence of the clinical impact of ^99m^Tc-MAA planning based on personalized dosimetry in large HCC has been provided in a multicentre randomized study comparing standard administration versus ^99m^Tc-MAA-based personalized dosimetry. Statistically significant increases of the response rate and the median OS (26.7 months versus 10.7 months, *p* = 0.012) were observed in the personalized dosimetry arm [67].

Despite the above-mentioned limitations, ^99m^Tc-MAA is currently the only method available for treatment planning for ^90^Y-microspheres.

Because the isotope ^166^Ho also emits gamma rays, the administration of a scout dose (250 MBq divided among injection positions; ±3 million particles) of ^166^Ho-microspheres is a viable alternative to ^99m^Tc-MAA. It has been shown to be safe [11] and to give a more accurate assessment of the lung shunt fraction [12]. Moreover, a scout dose of ^166^Ho-microspheres has also been shown to have a superior predictive value in comparison with ^99m^Tc-MAA for the prediction of intrahepatic of ^166^Ho-microsphere activity distribution [68]. Using the same microspheres for simulation and therapy, at least, the intrinsic problem of particle difference is overcome. Nevertheless, even the ^166^Ho-microspheres scout dose gives uncertain tumour absorbed dose prediction (95% C.I. of about ±100 Gy), indicating that, with the present available devices, the discrepancy between prediction and treatment is an intrinsic feature of radioembolization.

It is important to emphasize that in order to use ^99m^Tc-MAA or ^166^Ho-microsphere scout dose for intrahepatic dosimetry, the surrogate used (^99m^Tc-MAA or ^166^Ho-microspheres) and ^90^Y/^166^Ho microspheres should be injected in the same angiographic position, minimizing arterial spasm and the influence of vessel bifurcations, and injected slowly (20–30 s), in order to mimic microsphere infusion [67, 69]. Minimizing arterial spasm includes avoiding (whenever technically possible) coil embolization and favouring (whenever technically possible) the use of floppy catheters [67].

When ^99m^Tc-MAA (or ^166^Ho-microspheres scout dose) is used to plan the treatment with personalized dosimetry, a minimal pretherapy visual quality control is required evaluating the concordance between the ^99m^Tc-MAA tumour coverage and the CT or MRI tumour vascularity. Large discrepancies mean that the simulation was not accurate (e.g. influence of a bifurcation, spasm) and may be reconsidered.

To optimise the prediction of microsphere distribution, treatment should be performed timely after the diagnostic work up, ideally within 15 days.

## Treatment planning

A subset of different clinical scenarios has been described, depending on the tumour burden [69]. The following sections provide definitions of clinical scenarios and general dosimetry recommendations for the respective cohorts.

### Radiation segmentectomy

In patients with liver malignancy limited to ≤2 liver segments, higher absorbed doses to the perfused target volume can be administered. This leads to high response rates and long PFS [70–72], potentially superior to chemoembolization [73]. The risk is mitigated by the small volume of the irradiated liver. Therefore, it may also entail a viable treatment strategy for patients with a worse liver function. Limited data is available on patients with liver tumours other than HCC. Yet, the available literature suggests that the same principles can be applied [71], but further research is warranted.

### Radiation lobectomy

In some patients with unilobar disease, future liver remnants are insufficient to allow for resection. Unilobar treatment allows for higher absorbed doses, since part of the liver remains untreated, aiming for tumour control. Additionally, contralateral lobar hypertrophy can be induced, with the potential of bridging previously ineligible patients to resection with curative intent. Radiation lobectomy is also a feasible strategy in downstaging/bridge-to-transplant settings and includes a biologic test of time which may help identify patients most likely to benefit from resection. Hepatobiliary scintigraphy may be considered to assess future liver remnant function at baseline and during follow-up.

### Lobar disease with/without PVT

Patients with unilobar disease, who are not amenable for curative surgery because of portal hypertension, cirrhosis, PVT, extrahepatic disease or clinical performance, may be treated with a palliative intent. It is advised to use dosimetry for activity planning, aiming for a sufficient tumour absorbed dose, while keeping the absorbed dose to functional liver tissue below safety limits. Since only part of the liver is treated, higher absorbed doses to functional liver tissue may be acceptable to optimize the tumour absorbed dose. Hepatobiliary scintigraphy may be used to assess the differential function of the treated and non-treated parts of the liver. Targeting of existing PVT is fundamental for treatment success and should be evaluated prior to treatment [74].

### Bilobar disease

Patients in whom extensive disease precludes radiation segmentectomy or lobectomy, activity has to be planned with the aim to achieve high tumour absorbed doses with tolerable absorbed doses to functional liver tissue. High tumour absorbed doses have been shown to be correlated with treatment response, whereas high absorbed doses to functional liver parenchyma have been shown to increase the risk for radioembolization-induced liver disease (i.e. REILD). Sequential treatment of both liver lobes may be considered in patients with a dismal balance between dosimetry (i.e. absorbed dose to functional liver) and patient characteristics (e.g. liver cirrhosis, liver volume < 1.5 l, elevated bilirubin). In these cases, the typical interval ranges from 6 weeks to 3 months. A shorter interval may decrease the chance of interval progression but may increase the chance of cumulative toxicity. A longer interval may increase interval recovery but at the potential cost of interval progression. Treatment strategy should take these considerations into account, on an individual patient basis. Because of the documented variability of predictive dosimetry based on ^99m^Tc-MAA SPECT/CT, a sequential lobar treatment has the advantage that the prescribed activity of the second treatment can be adjusted based on the true ^90^Y-PET/CT based dosimetry of the first treatment. In this setting, shorter interval between treatments is acceptable. Hepatobiliary scintigraphy may be used to assess overall liver function.

## Dosimetry

Dosimetry can be performed using a single compartment model, a multi-compartment model or a method using a voxel-based approach. In the single compartment model, there is no distinction between the tumour and the normal liver parenchyma, and a mean dose is evaluated for the perfused volume. In the multi-compartment model, doses are evaluated separately for the tumour and the normal perfused liver. In voxel-based dosimetry, dosimetry is evaluated for each reconstructed voxel. Optimization of radiotherapeutic exposure, indicated in article 56 of the EU Council Directive 2013/59 and advised by a recent EANM position paper [75], requires a separate evaluation of target and nontarget tissues (multi-compartment dosimetry).

### Single versus multi-compartment versus voxel-based dosimetry

An obvious limitation of the single compartment model (as advocated for the standard use of glass ^90^Y-microspheres and ^166^Ho-microspheres and which can be called standard dosimetry) is that the actual spatial dose distribution of an individual patient accessible with the present scanner spatial resolution is neglected. In general, these methods seek to prevent overdosing to the functional liver parenchyma (and lungs), minimizing the occurrence of radioembolization-induced liver disease. As a consequence, the resulting prescribed activities are likely curbed by toxicity limitations of the most vulnerable patients and the occurrence of patients with a highly unfavourable dose distribution. This is thought to result in underdosing in some patients and overdosing in others.

In the multi-compartment model, a mean dose is individually evaluated for each compartment (tumour, normal liver and lung tissue). By doing so, it allows for the selection of a prescribed activity that maximizes the dose to the tumour tissue while not exceeding toxicity thresholds for the other two compartments. However, as a limitation, it does not consider the heterogeneity of the dose distribution in each compartment.

The respective compartments are usually segmented on an anatomical imaging modality (e.g. contrast-enhanced CT) and registered to the reconstructed tomographic distribution of a functional modality (e.g. SPECT thresholding). In the partition model [76], the activity distribution over the compartments is described by the tumour-to-normal tissue ratio (T/N ratio), expressed as.

$$\frac{T}{N}$$= $$\frac{A_{tumour}\left[\mathrm{MBq}\right]\ }{{\mathrm{M}}_{tumour}\ \left[\mathrm{kg}\right]}$$ / $$\frac{A_{normal\ liver}\ \left[\mathrm{MBq}\right]\ }{M_{normal\ liver}\left[\mathrm{kg}\right]}$$,

where *A* and *M* indicate the activity in and the mass of the tumour (T) and functional liver tissue (*N*) compartments.

The T/N ratio lays the foundation to calculate the desired treatment activity:

$$A(GBq)=\frac{D(Gy)\times \left(\left[\frac{T}{N}\times {M}_{tumour}\ \left[ kg\right]\right]+{M}_{liver}\left[ kg\right]\right)}{CF\times \left(1- lung\ shunt\ fraction\right)},$$

with CF being the absorbed dose conversion factor [CF(^90^Y) = 49.67 J/GBq; CF(^166^Ho) = 14.85 J/GBq].

Earlier implementations of this model had the limit of evaluating only the absorbed dose averaged over many lesions. A more general method capable of providing individual mean lesion absorbed dose evaluation is available [77].

Voxel-based dosimetry allows for the expression of (estimated) dose gradients and non-homogeneities on a small spatial scale, somewhat similar to external beam radiotherapy (EBRT). This contrasts with multi-compartment models, where dose estimates are averaged over each compartment. By including this spatial dimension, voxel-based dosimetry potentially provides a link to the rich EBRT literature on dose–effect relationships, which can be used for planning and outcome assessment. The usefulness of voxel dosimetry in nuclear medicine therapy is under debate [78]. In radioembolization, no study was able to demonstrate its superiority over the mean dose approach [77, 79, 80]. Many software solutions for dosimetry analyses, such as Simplicity™, Velocity Rapidsphere™, or Qsuite™, are now available [50].

## Activity calculation and treatment planning

Methodological details on the various technical aspects of dosimetry are reported in the recent EANM 90Y microspheres dosimetry guideline [81]. These guidelines do not apply to 166Ho microspheres, yet in the absence of dedicated guidelines, some of the underlying concepts may be applicable to treatment with 166Ho microspheres.

Whenever possible, multi-compartment dosimetry should be used. Whenever possible means that tumour segmentation is feasible and the clinical data support tumouricidal doses and maximum tolerated dose for the product used, the tumour histology and the indication (curative/palliative).

When multi-compartment dosimetry is not possible (e.g. infiltrative lesion, no/insufficient clinical dosimetry data available), simple single compartment dosimetry applied to the whole liver is conservative and should be used. Approaches described in the instruction for users of each product (e.g. single compartment dosimetry) can be used from a legal point of view, but they do not optimize treatment.

Although the current data on multi-compartment dosimetry indicate a dose–response relationship, reported dose thresholds vary and are mostly derived from retrospective studies [82]. The following sections and Tables 2, 3 and 4 provide an overview of dose thresholds that can be used for orientation, including the level of evidence supporting them. The reported data will be subject to change. Since most of the data was derived from non-comparative, retrospective analyses of dose–effect relationships, these preliminary data are very heterogeneous. Differences exist in terms of segmentation methods/software used, pre- versus post-treatment analysis, response criteria and reported dose metric (e.g. median/mean of response category, ROC analysis, most sensitive/specific for response). The reported numbers are based on current recommendations and reported literature; it is advised to read this literature before implementing dosimetry-based treatment planning in clinical practice.

**Table 2:** Absorbed dose recommendations for ^90^Y glass microspheres and the respective level of evidence (LOE)

	Single compartment		Multi-compartment		
Clinical scenario	Perfused volume dose	LOE	Normal liver dose	Tumour dose	LOE
HCC					
Segmentectomy	> 400 [83]	3	*Not applicable*		
Lobectomy	> 150 if whole liver dose <150 [67] 140–150 [84]	1* 3	≥ 88** [85] < 75 (range: 50/90***) [86]	≥ 205 [67] ≥ 250–300****	3
Unilobar	> 150 if whole liver dose <150 [67] 80–150 [61, 74]	1* 3	< 120** if HR < 30% [67] < 75 (range: 50/90***) [86]	≥ 205 [67] ≥ 250–300****	1* 3
Bilobar	80–150**** [13, 69, 87]	1, 4	< 50/90*** [86]	≥ 205 [62]	3
ICC					
Segmentectomy	> 400 [60]	4	*Not applicable*		
Lobectomy	140–150	4	< 75 (range: 50/90***)	≥ 260 [88]	3
Unilobar	80–150 [89]	3	< 75 (range: 50/90***)	≥ 260 [88]	3
Bilobar	80–150 [89]	3	< 75 (range: 50/90***)	≥ 260 [88]	3
mCRC					
Segmentectomy	> 400 [90]	3	*Not applicable*		
Lobectomy	140–150	4	< 75 (range: 50/90***)	≥ 189 [91]	3
Unilobar	80–150 [92]	3	< 75 (range: 50/90***)	≥ 189 [91]	3
Bilobar	80–150 [92]	3	< 75 (range: 50/90***)	≥ 189 [91]	3

*HR*, hepatic reserve, i.e. untreated liver fraction

*In patients comparable to the DOSISPHERE-01 [67] study population (Child-Pugh A, large lesions, at least 30% of hepatic reserve)

**Dose to the normal perfused liver, based on the first treatment

***Dose to the whole normal liver. In HCC patients with total bilirubin levels >1.1 mg/dl, an upper threshold of 50 Gy should be used; in patients with total bilirubin levels <1.1 mg/dl, the whole normal liver dose should be kept below 90 Gy. Data are derived from unilobar treatments without prior RE only. Since these thresholds have been established in mostly cirrhotic HCC patients, they can be considered safe for non-HCC patients; however, caution is warranted particularly in ICC patients with underlying cirrhosis and after chemotherapy

****For large lesions [67]

**Table 3:** Absorbed dose recommendations for ^90^Y resin microspheres and the respective level of evidence (LOE)

	Single compartment		Multi-compartment		
Clinical scenario	Perfused volume dose	LOE	Normal perfused liver dose	Tumour dose	LOE
HCC					
Segmentectomy	> 150 [93]	4	*Not applicable*		
Lobectomy	*Not recommended*		> 70 [93]*	≥ 100–120 [93]	4
Unilobar			< 40 [93]	≥ 100–120 [65]	3 4
Bilobar			< 30**/40 [93]	≥ 100–120 [65]	3 4
ICC					
Segmentectomy	> 150 [93]	4	*Not applicable*		
Lobectomy	*Not recommended*		> 70 [93]	≥ 100–120 [94]	3 4
Unilobar			< 40 [93]	≥ 100–120 *** [94]	3 4
Bilobar			< 30**/40 [93]	≥ 100–120 *** [94]	3 4
mCRC					
Segmentectomy	> 150 [93]	4	*Not applicable*		
Lobectomy	*Not recommended*		> 70 [93]	> 100 **** [93]	4
Unilobar			< 40 [93]	> 100 **** [95]	3 4
Bilobar			< 30**/40 [93]	> 100 **** [95]	3 4

Modified from Levillain et al. [93]

*Dose to the normal perfused liver with a hepatic reserve of >30%

**In pretreated patients or those with compromised liver function

***Longer OS for patients treated with a partition model-derived mean tumour dose of 86 Gy vs. BSA-derived tumour dose of 38 Gy

****Tumour absorbed doses >100 Gy have been associated with higher rates of metabolic complete response, whereas a lower threshold of >40–60 Gy predicted metabolic partial response

**Table 4:** Absorbed dose recommendations for ^166^Ho microspheres and the respective level of evidence (LOE)

	One-compartment		Multi-compartment		
Clinical scenario	Perfused volume dose	LOE	Whole normal liver dose	Tumour dose	LOE
HCC					
Segmentectomy	60	3	*Not applicable*		
Lobectomy	60	3	< 60	*Not available*	4
Unilobar	60	3	< 60	*Not available*	4
Bilobar	60	3	< 40***	*Not available*	4
ICC					
Segmentectomy	60	3	*Not applicable*		
Lobectomy	60	3	< 60	> 150*	3–4
Unilobar	60	3	< 60	> 150*	3–4
Bilobar	60	3	< 40*** [96]	> 150* [96]	3–4
mCRC					
Segmentectomy	60	3	*Not applicable*		
Lobectomy	60	3	< 60	> 90** [97]	3
Unilobar	60	3	< 60	> 90** [97]	3
Bilobar	60	3	< 40***	> 90** [97]	3

*LOE*, level of evidence

*Based on median tumour absorbed dose for stable disease in a mixed population

**Based on 100% sensitivity for response

***Up to 60 Gy in patients with more favourable liver function

Dosimetric planning driven mainly by tumour dose has two major weak points: first, for ^90^Y microspheres, the MAA lesion dose prediction may have an unacceptably large discrepancy with the actual therapeutic dose. Second, even a post-therapy ^90^Y PET dose above the proposed thresholds cannot guarantee response due to the overlap in dose distribution of responding and non-responding lesions. Therefore, in patients in whom level 1 evidence is not available [67], the administration of the maximum tolerable whole normal liver dose with a predicted tumour dose above the efficacy threshold constitutes an alternative driving criterion. Tumour dose is anyhow of outstanding importance in planning, since we do have level 1 evidence [60] that it prolongs median OS. In patients, in whom sufficient tumour doses cannot be reached without exceeding the proposed whole normal liver dose, alternative treatments may be considered.

Despite being outside of the recommendations provided in the IFU, doses >150 Gy to the whole liver have been shown to be safe [86], if the whole normal liver dose was <50/90 Gy. Due to lacking data (especially on OS), a general recommendation cannot be given.

### Resin ^*90*^Y-microspheres

The body surface area (BSA)-based method, initially advocated for resin ^90^Y-microspheres, was based on the observation that BSA correlates with liver volume in the healthy population [98]. The lack of personalization of activity prescription according to the true liver and tumour volumes of the patient, together with concerns about efficacy of this prescription method after several negative multicentre trials implementing this method (e.g. SIRFLOX, FOXFIRE, SARAH), induced further research on the absorbed dose–response relationship. The results lead to the recently published recommendation of a multidisciplinary expert panel to use the 3-compartimental partition model (or a voxel-based dosimetry method) and no longer the BSA-method or one-compartment approaches for activity prescription [93].

### Glass ^*90*^Y-microspheres

For glass ^90^Y-microspheres, the absorbed dose of a compartment (e.g. lung, tumour, normal perfused liver) is calculated using the simplified MIRD formula:

$$D\left[ Gy\right]=\frac{A\left[ GBq\right]x\ 50\left[\frac{J}{GBq}\right]}{M\left[ kg\right]}$$

where *D* = absorbed dose in the selected compartment, *A* = activity contained in the selected compartment and *M* the mass of this compartment.

The compartment mass may be determined using either CT, MRI, PET or ^99m^Tc-MAA SPECT.

Lung dose is calculated, assuming a lung mass of 1 kg, using the following MIRD formula:

$$DLung\left[ Gy\right]= injected\ Activity\left[ GBq\right]\kern0.5em \times LSF\times 50$$

The maximal tolerated dose is 30 Gy for one treatment and 50 Gy for repeated treatments.

The activity calculation is based on the desired mean absorbed dose to the target mass (tumour, perfused liver, normal liver), following

$$A\left[ GBq\right]= Desired\ dose\left[ Gy\right]\times \frac{Mtarget\left[ kg\right]\ }{50\ \left[J/ GBq\right]}$$

It has to be underlined that reported values for multi-compartment dosimetry (personalized dosimetry) were obtained for the use of glass microspheres on week 1 post-calibration, usually between 2- or 4-day post-calibration. No dosimetric data are available for a use on week 2 post-calibration.

### ^*166*^Ho-microspheres

Currently, activity calculation for ^166^Ho-microspheres is based on a method comparable to the Medical Internal Radiation Dose (MIRD) method used for glass ^90^Y-microspheres, as described above. The absorbed dose in Gy delivered by beta rays by 1 GBq in 1 kg tissue is 15.87 Gy for ^166^Ho, under the assumption of homogenous distribution in the target volume and absorption of all energy within that volume. The absorbed dose from gamma rays is relatively negligible. The formula for the prescribed activity is based on a 60 Gy average absorbed dose to the whole liver, following

$$A\left[ GBq\right]=\frac{Desired\ dose\left[ Gy\right]\times Mtarget\left[ kg\right]\ }{15.87\ \left[J/ GBq\right]}$$

According to current instructions for use, the average absorbed dose to the perfused volume may exceed 60 Gy, as long as the average absorbed to the whole liver does not exceed 60 Gy. Multi-compartment modelling is advised to optimize tumour absorbed doses while keeping absorbed doses to functional liver tissue within safety limits.

## Administration

For resin ^90^Y-microspheres, a typical treatment consists of injecting about 40–80 million ^90^Y-microspheres. Given the higher embolic load, no blind infusions should be performed. The catheter must be placed well distal (> 3–4 cm) to the gastroduodenal artery and any other artery that is supplying blood to the gastrointestinal system. The microspheres are delivered slowly at a rate of no more than 5 ml/min, as rapid delivery may cause reflux. The use of 5% glucose solution should be preferred over sterile water as it reduces stasis and thereby procedural patient discomfort [99]. During the procedure, the radiologist must repeatedly check the position of the catheter to ensure its position and continued forward flow. This is performed by injecting contrast medium through the left-hand port of the delivery set [100].

For glass ^90^Y-microspheres, a typical treatment consists of injecting 1.2–8 millions of glass ^90^Y-microspheres. The volume of saline solution required to infuse a vial is low (typically 20–60 ml). Furthermore, given the low number of microspheres infused, the entire vascular bed is never completely saturated and continuous fluoroscopic guidance during the infusion is not necessary. A complete infusion usually requires 20–60 ml and 5 min and should be performed with slow hand injection under free breathing.

For ^166^Ho-microspheres, the same administration system is used for both the scout dose and the treatment dose. A typical treatment consists of 20–30 million microspheres. Given the embolic load (albeit less compared with resin ^90^Y-microspheres), no blind infusions should be performed. The catheter must be placed well distal (> 3–4 cm) to the gastroduodenal artery, and any other artery that is supplying blood to the gastrointestinal system. The microspheres are delivered slowly at a rate of no more than 5 ml/min, as rapid delivery may cause reflux. Instead of 5% glucose, ordinary saline is used. During the procedure, administration of microspheres and contrast agent can be alternated by rotation of the dial between the administration and the contrast position, to check the catheter position and continued forward flow.

## Post-treatment imaging

For ^90^Y-microspheres, routine ^90^Y PET, planar scintigraphy and bremsstrahlung single-photon emission computed tomography with integrated computed tomography (SPECT/CT) can be considered for post-treatment imaging. Treating physicians should confirm sufficient tumour uptake, acceptable pulmonary absorbed dose and the absence of visceral ^90^Y concentration, in line with the pre-therapeutic ^99m^Tc-MAA SPECT/CT.

Despite the low amount of positron emission (31.86 ± 0.37 × 10^−6^), due to its high sensitivity and available time-of-flight (TOF) information, PET/CT quantitative imaging and dosimetry of ^90^Y-microspheres are feasible [101, 102]. In addition, PET/CT allows for a superior quantification and spatial resolution compared with bremsstrahlung SPECT/CT [5]. In patients with a considerable lung shunt fraction on pre-treatment ^99m^Tc-MAA scintigraphy, image acquisition should comprise the whole liver and lungs. Post-treatment planar imaging covering the lung region is advisable to identify unexpected lung shunt and complications that can be investigated more in detail with 3D modality [103, 104]. In the absence of a clinically relevant lung shunt fraction, coverage of the lungs is not necessary. With present PET/CT technology (3D acquisition and < 600 ps TOF resolution), to account for the low true-coincidence rate, emission times of at least 15 min (preferably more) are recommended [5, 105].

Due to the gamma-emitting properties of ^166^Ho-microspheres, post-treatment imaging can be performed by virtue of SPECT [106], ideally in conjunction with additional low-dose CT.

Upon decay, the isotope ^166^Ho emits several gamma photons, most of which are 81 keV (abundance 6.7%), 1379 keV (0.9%) or 1581 keV (0.2%). To quantify ^166^Ho accurately, the SPECT detector has to be set to register photons with an energy window of 7.5% around the photon peak of 81 keV. However, bremsstrahlung photons and other high-energy photons cause image-degrading effects because of scattering. Although this may limit accurate activity quantification, using a down-scatter window and a medium-energy collimator can reduce these effects. Immediately after administration, when the activity in the scanner is above approximately 1420 MBq (i.e. dependent on the SPECT/CT system), there is an excess of gamma photons that significantly increases detector dead time [106]. Dependent on the amount of administered activity, it is advised to perform post-treatment ^166^Ho SPECT/CT between 2 and 5 days after treatment.

Because of its paramagnetic properties, ^166^Ho can also be visualized and quantified using MRI techniques. MRI is independent of administered activity; however, because of artefacts, it is limited to tissue without air and metal. MRI has a higher resolution than SPECT/CT and takes less time. The presence of ^166^Ho accelerates the decay of the T2 vector of tissue. A linear relationship exists between T2* times and ^166^Ho concentration. This relationship, called the relaxivity (R2*), depends on the strength of the main magnetic field of the scanner and the ^166^Ho content.

The feasibility of MRI quantification of ^166^Ho-microspheres was validated in patients using SPECT/CT as a reference standard [107]. The recovery of ^166^Ho-microspheres in the liver using MRI proved to be sufficient (i.e. > 95%) for dosimetry purposes in patients without local surgical clips. A downside of the paramagnetic effects of ^166^Ho-microspheres is that its susceptibility artefacts obscure gadolinium enhancement on follow-up scans, which can potentially mask enhancing viable residual lesions.

## Facility and personnel

The facilities required will depend on national legislation for the emission of beta- (^90^Y-microspheres) and beta- and gamma-emitting (^166^Ho) therapeutics. If required by law, the patient should be admitted to an approved isolation facility comprising an appropriately shielded room and en suite bathroom facilities. The radiation field at 1 m from the patient’s abdomen immediately after administration is 1.14 μSv/h/GBq for ^90^Y microspheres [108]. This gives 3 μSv/h for typical 2.6 GBq glass microsphere administration, and 1.8 μSv/h for 1.6 GBq resin microspheres. For ^166^Ho, external exposure at 1-m spans from 8 to 60 μSv/h at 1 m [109].

The facility in which treatment is administered must have appropriate personnel, radiation safety equipment, procedures available for waste handling and disposal, handling of contamination, monitoring personnel for accidental contamination and controlling contamination spread.

The administration of ^90^Y- and ^166^Ho-microspheres should be undertaken by trained medical staff with supporting physics and nursing staff. Clinicians involved in unsealed source therapy must be knowledgeable about and compliant with all applicable national and local legislation and regulations.

To summarize, the development and establishment of an interdisciplinary team (interventional radiology; medical, radiation and surgical oncology; transplant surgery; nuclear medicine; hepatology; medical physics; and radiation safety) is crucial to the success of the treatment.

## Staff exposure

The most critical steps of exposure are the microsphere injections and, eventually, the microsphere preparation in the radiopharmacy (activity measuring and aliquoting when required).

A comparative study has evaluated occupational exposure for glass and resin ^90^Y-microspheres [110]. Reported equivalent doses H_p_(10) are less than 2 μSv/GBq for both microspheres for preparation and injection. Reported finger exposure with ^90^Y glass microspheres is 14.0 ± 7.9 μSv/GBq for the operator injecting microspheres and 13.5 ± 5.2 μSv/GBq for the radiopharmacist measuring the vial activity. With ^90^Y resin microspheres reported finger exposure are 235.5 ± 156 μSv/GBq for the operator injecting microspheres and 295.2 ± 271.9 μSv/GBq for the radiopharmacist measuring the vial activity and aliquoting the vial.

Another group evaluated equivalent doses H_p_(10) and finger doses from ^166^Ho exposure [111]. Reported whole-body doses were less than 3 μSv/GBq. Maximum finger doses of 2.9 ± 0.2 × 10^3^ μSv/GBq and 2.5 ± 0.3 × 10^3^ μSv/GBq (2.9 ± 0.2 μSv/MBq and 2.5 ± 0.3 μSv/MBq) have been reported for the for preparation and injection of ^166^Ho microspheres, respectively.

It should be taken into consideration that the limited ring sensitivity to beta emissions from 166Ho and 90Y may lead to a severe underestimation of actual absorbed doses. To account for this, measured dose rates can be complemented by an assessment of theoretical dose rates using dedicated software.

## Patient information and instruction

Patients should receive both written and verbal information on the procedure prior to therapy. Depending on the respective country’s legislation, informed written consent from the patient should be obtained. Unless performed in a preoperative setting, patients should be told that this therapy is not likely to cure their disease and is a palliative treatment directed to their liver lesion(s). Patients must be informed of the potential side effects of therapy and alternative treatment options. Patients must be advised to reduce unnecessary radiation exposure and contamination to family members and the public. Written instructions should be provided where required.

## Radiation protection

Any significant medical conditions should be noted, and contingency plans made in case radiation precautions must be breached for a medical emergency. Concern about radiation exposure should not interfere with the prompt appropriate medical treatment of the patient. Dose rate to the workers should be monitored during the treatment. Written instructions should be provided where required. The required radiation protection attention is different depending on the used radionuclide. It is minimal with ^90^Y since the external dose rate is low for all products used. With ^166^Ho, a significantly higher exposure rate after treatment should be considered. For all three products, substantial skin doses may occur in case of contamination during preparation or administration. After the treatment, patients should avoid pregnancy for at least 4 months. In reality, it is unlikely that women of childbearing age will be eligible for this therapy. Anyway, Gulec et al. [112] calculated that pregnancy shortly after a simulated treatment with ^90^Y-microspheres does not induce a relevant irradiation to the embryo. Long-lived radioisotope impurities have been observed and may be taken into consideration in the context of waste management [113]. They do not pose a radiation risk in the context of post-interventional surgery/biopsy.

## Side effects

^90^Y- and ^166^Ho-microspheres, common side effects (> 10% incidence; usually mild to moderate):

- Fatigue
- Abdominal pain
- Nausea
- Fever/cold chills
- Transitory elevation of liver enzymes
- Transitory decline in lymphocytes

^90^Y- and ^166^Ho-microspheres, possible severe adverse events (< 5%):

- Radioembolization-induced liver disease (i.e. hyperbilirubinemia, hypoalbuminemia, ascites, typically occurring 2–6 months after treatment, without evidence of disease progression)
- Non-target irradiation: radiation gastritis, gastrointestinal ulceration, upper gastrointestinal bleeding, pancreatitis, (radiation pneumonitis)

## Repeated treatment

Limited data on the feasibility of retreatment with radioembolization is available [114–117]. Based on the published literature, retreatment with radioembolization is feasible, has an acceptable toxicity profile and can be considered, especially in patients who responded to the first radioembolization treatment. Indications and contraindications described for primary treatment should be used for orientation. However, caution is warranted as most studies involved small and/or heterogeneous sample sizes. No published data on repeated treatment with multi-compartment dosimetry have been published yet. The cumulative absorbed doses to non-tumour liver tissue should be assessed at all times [69].

## Follow-up

Monitoring of side effects should take pre-treatment liver function (e.g. the presence/absence of cirrhosis) and the treatment protocol (e.g. absorbed dose, fraction of treated liver) into account. Generally speaking, first laboratory tests and clinical evaluations should take place around 2 to 4 weeks after treatment. Follow-up examinations should be scheduled according to the results.

The first follow-up cross-sectional imaging may be performed 3 months after radioembolization [69]. Response criteria purely based on lesion size (such as RECIST) ignore the occurrence of necrosis and decreased perfusion observed after local ablative treatment and have shown poor correlation with outcome parameters in HCC patients [118]. To account for this, modified RECIST (mRECIST) criteria rely on size assessment of the viable tumour instead [119]. Modified RECIST criteria have shown superiority in response assessment in HCC patients undergoing local treatment and should therefore be used [118, 120–122]. Less data is available on metastatic neuroendocrine neoplasms. Across studies, different response criteria were used [123]. RECIST response was significantly associated with OS; in hypervascular tumours, response assessment pursuant to mRECIST criteria may be beneficial [124, 125]. For ICC, size changes of the viable part of the tumour (as assessed by EASL, mRECIST or CHOI criteria) are associated with OS, whereas this correlation was not shown for conventional RECIST [126, 127]. In mCRC on the other hand, RECIST was used for response assessment in multiple RCTs [24, 30, 43] and correlated with OS, whereas mRECIST did not [128]. Additionally, in mCRC patients, FDG-PET may allow for earlier and improved response assessment compared with conventional imaging [40].

## Future directions 

Main challenges for future studies include defining and validating dose thresholds in different clinical scenarios. Additionally, prior studies using BSA- or uni-compartment-based approaches yielded negative results, the efficacy of radioembolization using multi-compartment dosimetry needs to be assessed in randomised controlled trials in comparison/ addition to standard of care treatment.

## Footnotes

1 ^99m^Tc-labelled human serum albumin (HSA) particles are not widely available but can also be used.

2 Planar and, possibly, single-photon emission computed tomography with integrated computed tomography (SPECT/CT)

3 Oral administration of sodium perchlorate before the MAA injection can be considered to prevent physiological uptake of free ^99m^Tc-pertechnetate in the stomach that can be mistaken for visceral shunting.

## Declarations

### Liability statement

This guideline summarises the views of the EANM Dosimetry, Oncology and Theranostic, Radiation Protection Committee. It reflects recommendations for which the EANM cannot be held responsible. The recommendations should be taken into context of good practice of nuclear medicine and do not substitute for national and international legal or regulatory provisions
